# Early detection of psoriatic arthritis in patients with psoriasis: construction of a multifactorial prediction model

**DOI:** 10.3389/fimmu.2024.1426127

**Published:** 2024-12-11

**Authors:** Chunxiao Wang, Sihan Wang, Liu Liu, Jiao Wang, Xiaoce Cai, Miao Zhang, Xiaoying Sun, Xin Li

**Affiliations:** ^1^ Department of Dermatology, Yueyang Hospital of Integrated Traditional Chinese and Western Medicine, Shanghai University of Traditional Chinese Medicine, Shanghai, China; ^2^ Institute of Dermatology, Shanghai Academy of Traditional Chinese Medicine, Shanghai, China; ^3^ Department of Dermatology and Venereology, National Clinical Research Center for Dermatologic and Immunologic Diseases of China, Peking University First Hospital, Beijing, China

**Keywords:** psoriatic arthritis, prediction model, web-based calculator, psoriasis, decision curve analysis

## Abstract

Psoriatic arthritis (PsA) affects approximately one in five individuals with psoriasis. Early identification of patients with psoriasis at risk of developing PsA is crucial to prevent poor prognosis. We established a derivation cohort comprising 1,661 patients with psoriasis from 49 hospitals. Clinical and demographic variables ascertained at hospital admission were screened using the Least Absolute Shrinkage and Selection Operator and logistic regression to construct a prediction model and a new web-based calculator. Ultimately, six significant independent predictors were identified: history of unexplained swollen joints (odds ratio [OR]: 5.814, 95% confidence interval [95% CI]: 3.304–10.117; p< 0.001), history of arthritis (OR: 3.543, 95% CI: 1.982–6.246; p< 0.001), history of unexplained swollen and painful fingers or toes (OR: 2.707, 95% CI: 1.463–4.915; p = 0.001), nail involvement (OR: 1.907, 95% CI: 1.235–2.912; p = 0.003), hyperlipidemia (OR: 4.265, 95% CI: 0.921–15.493; p = 0.042), and prolonged topical use of glucocorticosteroids (OR: 1.581, 95% CI: 1.052–2.384, p = 0.028). The web-based calculator derived from this model can assist clinicians in promptly determining the probability of developing PsA in patients with psoriasis, thereby facilitating improved clinical decision-making.

## Introduction

Psoriatic arthritis (PsA) affects approximately one in five patients with psoriasis (1), and a diagnostic delay of 6 months can lead to peripheral joint erosion and deterioration of physical function (2). Although significant advancements have been made in psoriasis treatment, early screening and preventive measures remain crucial for minimizing irreversible joint damage and improving long-term patient outcomes (3).

While factors such as obesity, nail pitting, and uveitis have been identified as potential predictors of the progression from psoriasis to PsA, existing screening tools often lack the precision needed to offer rapid and practical clinical guidance (4, 5). The limitations of these tools present a significant challenge in preventing the transition from psoriasis to PsA, emphasizing the need for more accurate and efficient predictive strategies.

Early diagnosis is essential for initiating interventions to prevent structural damage associated with PsA. Developing convenient, personalized prediction tools is critical not only for raising awareness about the importance of early intervention, but also for enabling healthcare providers to deliver targeted care. These tools can optimize the allocation of healthcare resources, ultimately leading to better management of PsA risk and improved patient outcomes. The primary objective of this study was to develop and validate a risk prediction model for PsA in patients with psoriasis with the goal of preventing disease progression and mitigating the long-term effects of structural damage.

## Methods

### Data sources and processing

To describe the development and validation of our multivariable prediction model, we followed the Transparent Reporting of a Multivariable Prediction Model for Individual Prognosis or Diagnosis (TRIPOD) statement (**Supplementary Table S1**). This study was approved by the Ethics Committee of the Yueyang Hospital of Integrated Traditional Chinese and Western Medicine (No: 2021-127; November 26, 2021). Informed consent was obtained using a web-based form, and all data were de-identified.

The China Psoriasis Diagnostic and Treatment Center platform (http://www.psocenter.cn/) was established in 2019 and includes observational, prospective, and retrospective studies. These studies investigated various aspects of psoriasis, including its burden, specific types, drug efficacy and safety, disease progression, comorbidity characteristics, and psychological impact. All the participating units underwent qualification audits. Collaborating with the platform, we collected a derivation cohort comprising 1,661 patients with psoriasis from 49 hospitals nationwide between June 24, 2020, and June 30, 2021, to develop a risk prediction model. Data from 707 patients with psoriasis collected between July 1, 2021, and December 30, 2021, were used for external validation. Patients diagnosed with PsA at baseline by a rheumatologist were excluded. The follow-up period was 1 year, and the primary outcome was the development of PsA within a one-year follow-up period. The patients were classified into two distinct groups based on whether they developed PsA. Missing data were analyzed using multiple imputations (**Supplementary Figure S1**).

### Case definition

During each follow-up visit, rheumatologists evaluated the patient’s muscles and bones. If clinical indications suggested abnormalities, further laboratory and imaging tests were performed to confirm whether the patient had developed PsA.

For patients suspected of having arthritis based on their clinical symptoms, rheumatologists performed further imaging examinations. These include:

1. X-rays: To identify any changes in bone, such as erosions or new bone formation, which can be indicative of PsA.
2. Ultrasound: To assess joint inflammation, synovitis, enthesitis, and dactylitis, which are common in PsA, in real time.
3. MRI: To provide detailed images of the joints and soft tissues, revealing inflammation and structural damage not visible on X-rays.
4. Laboratory tests: Including C-reactive protein (CRP), erythrocyte sedimentation rate (ESR), rheumatoid factor (RF), and anti-cyclic citrullinated peptide antibodies (ACPA).

### Potential predictive variables

The following potential predictive variables were collected at the time of initial admission: sex; age; duration of psoriasis; body mass index; marital status; educational level; smoking history; drug allergy; tumor history; photoallergy; history of unexplained joint swelling (early, potentially transient joint inflammation, which might not yet meet the diagnostic criteria for arthritis), arthritis (diagnosed with arthritis by a rheumatologist, but not including rheumatoid arthritis.), unexplained heel pain, and unexplained painful swelling of fingers or toes; family history of psoriasis; nail, scalp, or palmoplantar involvement; body surface area (BSA) involved; psoriasis area and severity index (PASI); pre-existing cardiovascular disease, type-1 or type-2 diabetes, hyperlipidemia, and hyperuricemia; abnormal transaminases; use of topical vitamin D3 derivatives, glucocorticosteroids, and tretinoin, oral methotrexate, and use of biologic therapies; satisfaction with treatment; and history of atopic dermatitis, tuberculosis, fatty liver disease, gastric ulcer, rheumatoid arthritis, and allergic rhinitis.

The following conditions were included in our study to explore their potential role as risk factors for PsA and provide a comprehensive understanding of the disease burden in patients with psoriasis.

Atopic Dermatitis (AD): Atopic dermatitis and psoriasis share overlapping immune pathways. In some cases, patients with AD may exhibit a mixed inflammatory pattern of Th2/Th17 cells (6). In addition, both psoriasis and AD involve skin barrier dysfunction and disorders of the skin microbiota that may promote inflammation. By recording the history of AD, our study aimed to investigate whether these shared pathways contribute to the development of PsA in patients with psoriasis.

Tuberculosis (TB): Patients with psoriasis, especially those treated with immunosuppressive therapies (e.g., biologics and corticosteroids), are at an increased risk of tuberculosis reactivation (7). Chronic inflammation also correlates with a higher risk of TB, which may be relevant for patients with PsA owing to the inflammatory nature of the disease.

Gastric Ulcer: Although the direct association between gastric ulcers and PsA is unclear, long-term use of NSAIDs or corticosteroids in managing psoriasis and PsA may increase the risk of gastric ulcers. By monitoring this comorbidity, we can evaluate how management strategies contribute to gastrointestinal complications in patients with psoriasis (8).

Rheumatoid Arthritis (RA): Rheumatoid arthritis (RA) and psoriatic arthritis (PsA) are distinct joint diseases. Although RA is not directly linked to PsA, its presence suggests that an individual has a propensity to develop other non-psoriatic arthritic conditions.

Allergic Rhinitis: Similar to atopic dermatitis, allergic rhinitis is driven by Th2 responses and may share immune pathways with psoriasis and PsA. Studies have suggested a possible link between allergic rhinitis and an increased risk of autoimmune diseases. Including this comorbidity allowed us to explore whether Th2-driven immune activity plays a role in PsA development (9).

In summary, these comorbidities were recorded to assess their potential role as risk factors for PsA in psoriasis patients. Understanding these associations may facilitate early detection and provide insights into the immune mechanisms underlying PsA.

### Variable selection and model construction

**Figure 1:**
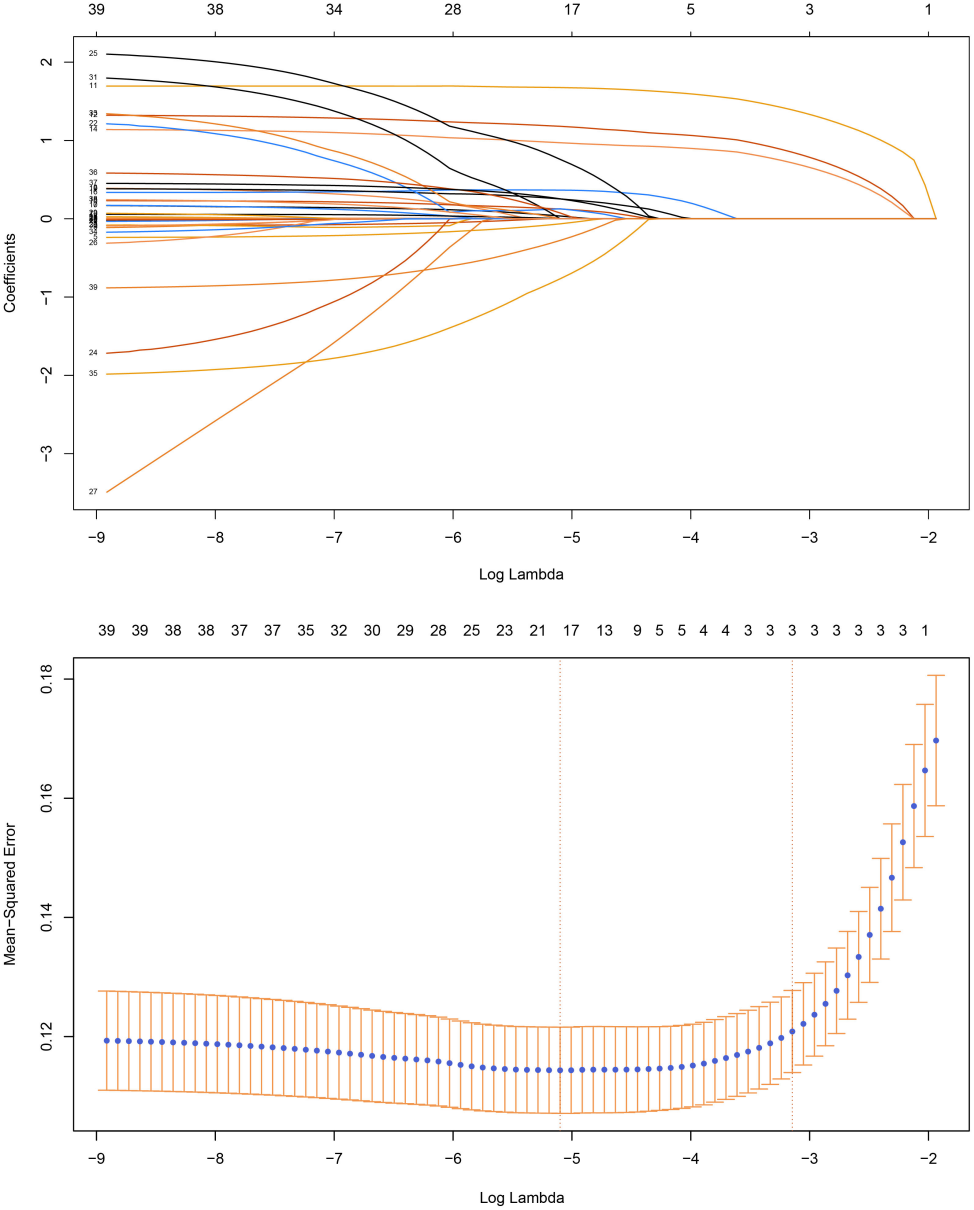
Least Absolute Shrinkage and Selection Operator (LASSO) Regression and 10-fold Cross-Validation.

A total of 39 candidate variables were included in the predictive model. Least Absolute Shrinkage and Selection Operator (LASSO) regression were used to reduce the dimensionality. LASSO regression compresses the variables by imposing a penalty function called “lambda,” simplifying the model and reducing multicollinearity and overfitting between the variables. As lambda increases, the coefficients of each variable are gradually reduced, and the unimportant variables are gradually compressed to zero, thus enabling variable selection. A 10-fold cross-validation was performed to select the best parameter (lambda). Finally, the optimal lambda corresponds to the 19 predictor variables indicated by the dotted line on the left side (**Figure 1**). Subsequently, these variables were entered into a multifactorial logistic regression model. Significant variables identified in the logistic regression results were used to construct a risk-prediction model and develop a web-based calculator.

### Evaluation of the prediction model

We used the area under the receiver operating characteristic curve (AUC) to quantify sample discrimination, where an AUC > 0.75 indicates a high degree of discrimination (10). Calibration was evaluated using a calibration curve based on 1,000 bootstrap samples and the *Hosmer–Lemeshow* test. The Hosmer–Lemeshow test p-value greater than 0.05 indicates that the predicted probability is not statistically different from the actual result, suggesting that the model has a good calibration (11). The Youden index, defined as the Youden index = true positive rate + true negative rate – 1. The Youden index can be used to calculate the optimal threshold probability for maximizing the benefits of the model. Decision curve analysis (DCA) and clinical impact curve (CIC) were implemented to validate the model’s net benefits and effectiveness.

All statistical analyses were performed using the R software (version 4.1.3; R Core Team [2022]. Vienna, Austria. URL: https://www.R-project.org/). Significance was set at p< 0.05.

## Results

### Characteristics of the cohort

The derivation cohort comprised 1,661 patients with psoriasis, 156 (9%) of whom were diagnosed with PsA at the 1-year follow-up visit. **Table 1** presents the demographic and clinical characteristics of the patients with or without PsA in the derivation cohort. The validation cohort consisted of 707 patients with psoriasis, 67 (9%) of whom were diagnosed with PsA during the 1-year follow-up visit. **Supplementary Table S2** presents the demographic and clinical characteristics of the external validation cohort.

**Table 1:** Demographic and clinical characteristics of patients in the derivation cohort.

Variables	Total (n = 1661)	PsA-yes (n = 156)
Sex, n (%)		
Female	640 (39)	53 (34)
Male	1021 (61)	103 (66)
**Age, Median (Q1, Q3)**	37 (31, 50)	41 (33, 54)
**Duration of psoriasis, Median (Q1, Q3)**	7 (3, 14)	10 (4, 18)
**BMI, Median (Q1, Q3)**	24.22 (21.97, 26.71)	24.34 (22.36, 26.52)
Married, n (%)		
Yes	1227 (74)	126 (81)
No	434 (26)	30 (19)
Education, n (%)		
Junior high	473 (29)	49 (32)
Senior high	588 (36)	56 (37)
Undergraduate	571 (35)	48 (31)
Smoking, n (%)		
No	1117 (67)	91 (58)
Yes	544 (33)	65 (42)
Drug allergy, n (%)		
No	1326 (93)	129 (93)
Yes	95 (7)	9 (7)
Tumor history, n (%)		
No	1639 (99)	153 (98)
Yes	22 (1)	3 (2)
Photoallergy, n (%)		
Yes	336 (20)	30 (19)
No	1324 (80)	126 (81)
History of unexplained swollen joints, n (%)		
No	1464 (89)	62 (41)
Yes	172 (11)	89 (59)
History of arthritis, n (%)		
No	1487 (91)	75 (50)
Yes	144 (9)	75 (50)
History of unexplained heel pain, n (%)		
No	1483 (91)	100 (66)
Yes	145 (9)	51 (34)
History of unexplained swollen and painful finger or toe, n (%)		
No	1473 (90)	69 (46)
Yes	159 (10)	81 (54)
Family history of psoriasis, n (%)		
No	241 (16)	33 (23)
Yes	1252 (84)	112 (77)
Nail involvement, n (%)		
No	1243 (77)	76 (51)
Yes	378 (23)	74 (49)
Scalp involvement, n (%)		
No	516 (31)	36 (23)
Yes	1136 (69)	118 (77)
Palmoplantar involvement, n (%)		
No	1344 (81)	102 (66)
Yes	307 (19)	53 (34)
Genital involvement, n (%)		
No	1410 (86)	108 (71)
Yes	229 (14)	44 (29)
**BSA, Median (Q1, Q3)**	11 (3.02, 30)	15.5 (3.25, 40)
**PASI, Median (Q1, Q3)**	8.4 (3.3, 16.9)	8.15 (2.7, 19.42)
Cardiovascular disease, n (%)		
No	1583 (95)	140 (90)
Yes	78 (5)	16 (10)
Type-1 Diabetes, n (%)		
No	1406 (>99)	111 (98)
Yes	4 (<1)	2 (2)
Type-2 Diabetes, n (%)		
No	1396 (98)	110 (98)
Yes	22 (2)	2 (2)
Hyperlipidemia, n (%)		
No	1406 (>99)	112 (99)
Yes	4 (<1)	1 (1)
Abnormal transaminases, n (%)		
No	1402 (>99)	108 (>99)
Yes	4 (<1)	0 (<1)
Atopic dermatitis, n (%)		
No	1271 (>99)	114 (>99)
Yes	2 (<1)	0 (<1)
Topical use of vitamin D3 derivatives, n (%)		
No	1193 (72)	113 (72)
Yes	468 (28)	43 (28)
Oral methotrexate, n (%)		
No	1561 (94)	147 (94)
Yes	100 (6)	9 (6)
Satisfaction with treatment, n (%)		
Highly satisfied	158 (10)	20 (13)
Satisfied	399 (24)	26 (17)
Ordinary	755 (45)	81 (52)
Dissatisfied	277 (17)	23 (15)
Extremely dissatisfied	72 (4)	6 (4)
Hyperuricemia, n (%)		
No	1408 (>99)	113 (>99)
Yes	1 (<1)	0 (<1)
Tuberculosis, n (%)		
No	1395 (>99)	109 (>99)
Yes	2 (<1)	0 (<1)
Fatty liver disease, n (%)		
No	1400 (>99)	108 (>99)
Yes	7 (<1)	0 (<1)
Gastric ulcer, n (%)		
No	1399 (>99)	108 (99)
Yes	3 (<1)	1 (1)
Rheumatoid arthritis, n (%)		
No	1264 (>99)	114 (>99)
Yes	2 (<1)	0 (<1)
Allergic rhinitis, n (%)		
No	1267 (99)	113 (99)
Yes	7 (1)	1 (1)
Topical use of glucocorticosteroids, n (%)		
No	961 (58)	79 (51)
Yes	700 (42)	77 (49)
Topical use of tretinoin, n (%)		
No	1453 (87)	133 (85)
Yes	208 (13)	23 (15)
Prior biologic therapy, n (%)		
No	1485 (90)	142 (93)
Yes	158 (10)	11 (7)

BMI, body mass index; BSA, body surface area; PASI, psoriasis area and severity index; PsA, psoriatic arthritis; PsA-no: Patients with psoriasis not diagnosed with psoriatic arthritis; PsA-yes, patients with psoriasis who were diagnosed with psoriatic arthritis.

### Predictive variable selection

After LASSO regression, the following 19 predictors were identified: age, duration of psoriasis, marital status, smoking history, history of unexplained joint swelling, arthritis, unexplained heel pain, unexplained swelling and pain in the fingers or toes, nail involvement, palmoplantar and genital involvement, BSA, presence of cardiovascular and fatty liver diseases, hyperlipidemia, rheumatoid arthritis, allergic rhinitis, prolonged topical use of glucocorticoids, and prior biological therapy.

The optimal model was selected after including these 19 variables in the logistic regression model. Ultimately, six statistically significant independent predictors were identified: history of unexplained swollen joints (odds ratio [OR]: 5.814, 95% confidence interval [95% CI]: 3.304–10.117; p< 0.001), history of arthritis (OR: 3.543, 95% CI: (1.982–6.246; p< 0.001), history of unexplained swollen and painful fingers or toes (OR: 2.707, 95% CI: 1.463–4.915; p = 0.001), nail involvement (OR: 1.907, 95% CI: 1.235–2.912; p = 0.003), hyperlipidemia (OR: 4.265, 95% CI: 0.921–15.493; p = 0.042), and prolonged topical use of glucocorticosteroids (OR: 1.581, 95% CI: 1.052–2.384, p = 0.028) (**Table 2**).

**Table 2:** Multivariate logistic regression model for predicting the development of psoriatic arthritis in patients with psoriasis.

Variables (-yes)	Beta	Wald	OR with 95% CI	*p-*value
(Intercept)	-3.614	390.396	0.027 (0.019–0.038)	<0.001
History of unexplained swollen joints	1.760	38.170	5.814 (3.304–10.117)	<0.001
History of arthritis	1.265	18.723	3.543 (1.982–6.246)	<0.001
History of unexplained swollen and painful finger or toe	0.996	10.411	2.707 (1.463–4.915)	0.001
Nail involvement	0.646	8.733	1.907 (1.235–2.912)	0.003
Hyperlipidemia	1.450	4.123	4.265 (0.921–15.493)	0.042
Topical use of glucocorticosteroids	0.458	4.844	1.581 (1.052–2.384)	0.028

OR, odds ratio; CI, confidence interval.

### Construction of the web-based calculator

**Figure 2:**
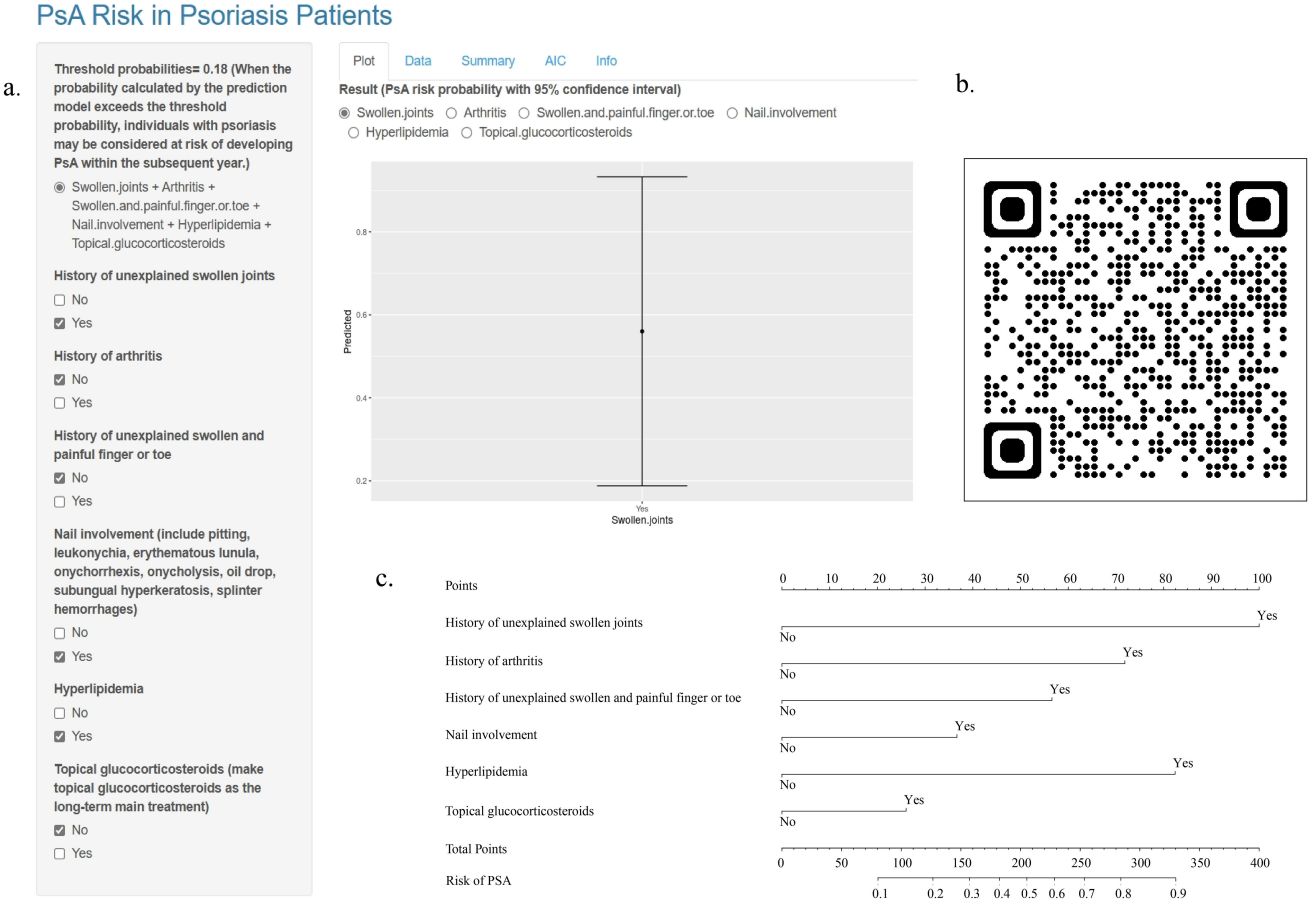
Online Web-Based Calculator and Common Nomogram for Predicting PsA in Patients with Psoriasis. **(A)** Webpage of an Online Web-Based Calculator (https://detection.shinyapps.io/PsA-Risk/). **(B)** QR Code for the Online Web-Based Calculator. **(C)** Common Nomogram of Prediction Model.

A prediction model and a web-based calculator were developed using logistic regression analysis. The web-based calculator (https://detection.shinyapps.io/PsA-Risk/) was designed to enable clinicians to input information regarding the six predictive variables and automatically calculate the likelihood (with a 95% CI) that a patient with psoriasis will develop PsA within 1 year. Additionally, a common nomogram was created as a reference (**Figure 2**).

### Performance of the prediction model

**Figure 3:**
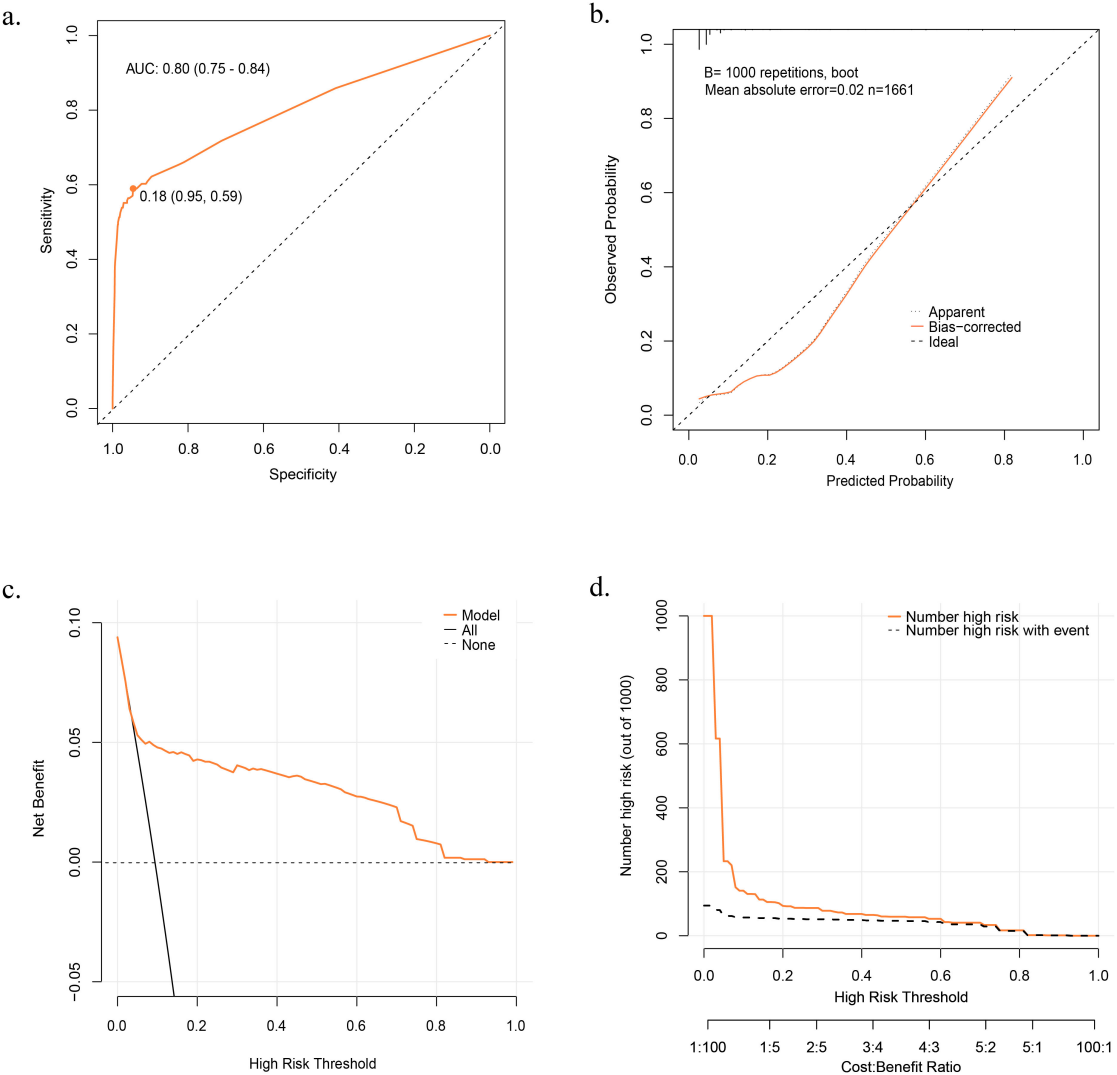
Evaluation of the Prediction Model. **(A)** Receiver Operator Characteristic (ROC) curve in Derivation Cohort. **(B)** Calibration Curve in Derivation Cohort. **(C)** Decision Curve Analysis (DCA) curve in Derivation Cohort. **(D)** Clinical Impact Curve (CIC) in Derivation Cohort. *Net benefit = true positive rate* - *(false positive rate* × *weighting factor)*. $Weighting\;factor=\frac{Threshold\;probability}{1-threshod\;probability}$ .

The prediction model demonstrated excellent discrimination, with AUCs of 0.80 (95% CI: 0.75–0.84) and 0.77 (95% CI: 0.70–0.84) when using the derivation and validation cohorts, respectively. Based on the Youden index, we identified potential optimal threshold probability points on a receiver operating characteristic (ROC) curve. In the derivation and validation cohorts, the optimal threshold probabilities were determined to be 0.18 (specificity = 0.95, sensitivity = 0.59) and 0.23 (specificity = 0.96, sensitivity = 0.54), respectively (**Figure 3A**; **Supplementary Figure S2**). When the probability calculated by the prediction model exceeds the threshold probability, individuals with psoriasis may be considered to be at risk of developing PsA within the subsequent year.

The model exhibited favorable performance in terms of calibration. The p-values from the *Hosmer–Lemeshow* test in the derivation and validation cohorts were 0.16 and 0.11, respectively. Additionally, the calibration curve based on 1,000 bootstrap resamples indicated an excellent goodness of fit for the model (**Figure 3B**).

DCA and CIC were plotted to assess the model’s clinical effectiveness and net benefits. In the DCA, the X-axis represents the threshold probability, whereas the y-axis represents the net benefit.


Net benefit=true positive rate−(false positive rate×weighting factor)

$$Weighting\;factor=\frac{Threshold\;probability}{1-threshod\;probability}$$

The orange line in the DCA represents the net benefit of the model for various threshold probabilities. Additionally, two unique lines are included in the figure: a horizontal dotted line representing a scenario in which no one is treated (resulting in a net benefit of zero, regardless of the threshold probability) and a black line illustrating the change in net benefit as the probability threshold is adjusted if everyone is treated, the two lines represent the two extremes. A higher net benefit indicates better model performance for a given probability threshold and the farther the orange curve is from the two unique lines, the better the clinical performance of the model (**Figure 3C**). In the CIC, the X-axis corresponds to the threshold probability, whereas the y-axis represents the number of high-risk individuals. The orange line in the CIC indicates the number of high-risk patients identified using the model at various threshold probabilities. The dashed black line represents the number of high-risk individuals who developed PsA at the different probability thresholds. The closer the solid orange and dashed gray lines are to each other, the better the predictive power of the model aligns with actual clinical outcomes (**Figure 3D**). The DCA and CIC curves provided insights into the actual clinical performance of the model under different threshold probabilities. The DCA curve demonstrated a favorable performance, indicating a net benefit even at higher risk judgment threshold probabilities. Meanwhile, the CIC curve illustrates that the model’s predictions increasingly align with actual clinical outcomes as the risk determination threshold probability exceeds 0.2. When the threshold probability exceeds 0.6, the predicted results are almost the same as the actual results, indicating strong clinical performance of the model.

## Discussion

The inclusion variables were a history of unexplained swollen joints, arthritis, unexplained swollen and painful fingers or toes, nail involvement, hyperlipidemia, and prolonged topical use of glucocorticosteroids. Previous studies have shown a close association between joint tenderness and swelling in patients with PsA (12, 13). Patients with nail involvement have a higher risk of systemic enthesitis, a key feature of PsA (14). Hyperlipidemia is associated with an increased risk of psoriasis and psoriasis with PsA. HDL cholesterol is significantly lower, and LDL is markedly higher in patients with psoriatic arthritis (15, 16). Dactylitis, characterized by swollen fingers or toes, is a common sign of PsA and an important diagnostic marker for early PsA detection (17). Although a limited number of studies have suggested a potential association between topical glucocorticosteroid use and PsA, the evidence remains inconclusive (18). The direct correlation between glucocorticosteroid use and PsA development in patients with psoriasis cannot be understood in isolation. Long-term use of topical glucocorticoids may reflect the severity of the patient’s condition rather than the direct cause of PsA development.

In this study, we developed and validated a clinical prediction model using six optimal predictors to predict the probability of developing PsA in patients with psoriasis. Our model performed well, exhibiting high discrimination and calibration in both the derivation and validation cohorts. The DCA and CIC curves further underscore the practical clinical utility of the model. In addition to utilizing the optimal threshold probability point (0.18) identified on the ROC curve, clinicians can leverage DCA and CIC curves to develop individualized medical plans tailored to local medical resources and patient-specific circumstances. By referring to the DCA and CIC curves, clinicians can adjust the risk determination threshold probability upwards, aligning model predictions more closely with actual patient outcomes. This approach conserves healthcare resources and maintains the net benefit of the model’s predictions. Moreover, the model passed the external validation, and both the internal and external validation AUCs were more significant than 0.75, indicating that the model had a high level of generalizability.

Compared with existing predictive models, particularly the PRESTO model developed by Gladman et al., which utilizes predictors such as nail pitting, morning stiffness, uveitis, and biologic therapy usage, our model exhibits superior performance (19). The PRESTO model reported AUCs of 72.3% for 1-year predictions and 74.9% for 5-year predictions, whereas our model achieved an AUC of 80% for 1-year predictions, with a specificity of 85% and a sensitivity of 78%. This enhanced predictive accuracy underscores the capability of our model to effectively identify patients at risk of developing PsA. Moreover, the limitations of the existing predictive models highlight the need for more comprehensive performance metrics. Our model’s enhanced predictive capability, operational simplicity, and adaptability to resource-limited settings make it a valuable tool for the early detection and intervention of PsA. By improving risk stratification, our model aimed to facilitate timely clinical interventions that could significantly alter the disease trajectory in patients with psoriasis.

Finally, we developed a web-based risk calculator to help clinicians determine the risk of developing PsA within a year in patients with psoriasis. This would enable clinicians to make better decisions. Users scan the QR code to enter the website, select variables from the six predictors according to their conditions, and obtain the corresponding PsA risk probability with a 95% confidence interval.

### Limitations

This study has some limitations. First, additional laboratory-related variables should be included. Second, the data used for the derivation and validation cohorts were obtained exclusively from Chinese patients. Therefore, the generalizability of our model may be limited when applied to populations outside of China. Finally, in our cohort, 9.48% (67/707) of patients with psoriasis developed PsA within 1 year. This rate is higher than that reported in other studies. For example, Eder et al. conducted a cohort study at the Toronto Psoriasis Cohort (2006–2014) and reported an annual PsA incidence of 2.7 per 100 psoriasis patients (95% CI 2.1–3.6), increasing to 3.2 per 100 when including suspected cases (5). Similarly, Lindberg et al. (2007–2017) in Sweden reported an incidence of 1.69 per 100 patient-years, rising to 5.49 per 100 patient-years in biologically treated patients (20). The higher incidence in our cohort may be due to the inclusion of more severe psoriasis cases, as nail involvement and higher PASI scores are strong predictors of PsA.

## Conclusions

In this study, we developed a clinical prediction model and web-based calculator based on six predictors to estimate the risk of developing PsA in patients with psoriasis. Our model provides clinicians with a valuable tool to assess the likelihood of developing PsA. This capability facilitates informed clinical decisions, enables tailored treatment strategies, and optimizes the use of medical resources.

## Data Availability

The original contributions presented in the study are included in the article/**Supplementary Material**. Further inquiries can be directed to the corresponding authors.

## Supplementary material

Supplementary Figure 1 Proportion of Missing Data in Derivation Cohort and Validation Cohort.

Supplementary Figure 2 Receiver Operator Characteristic (ROC) curve and Calibration Curve in Validation Cohort.
