# Supplementary figures and images for: Early detection of psoriatic arthritis in patients with psoriasis: construction of a multifactorial prediction model
Source: Front Immunol. 2024 Dec 11;15:1426127. doi: 10.3389/fimmu.2024.1426127 (PMC11668630; doi:10.3389/fimmu.2024.1426127)

Fig. S1. Proportion of Missing Data in Derivation Cohort and Validation Cohort

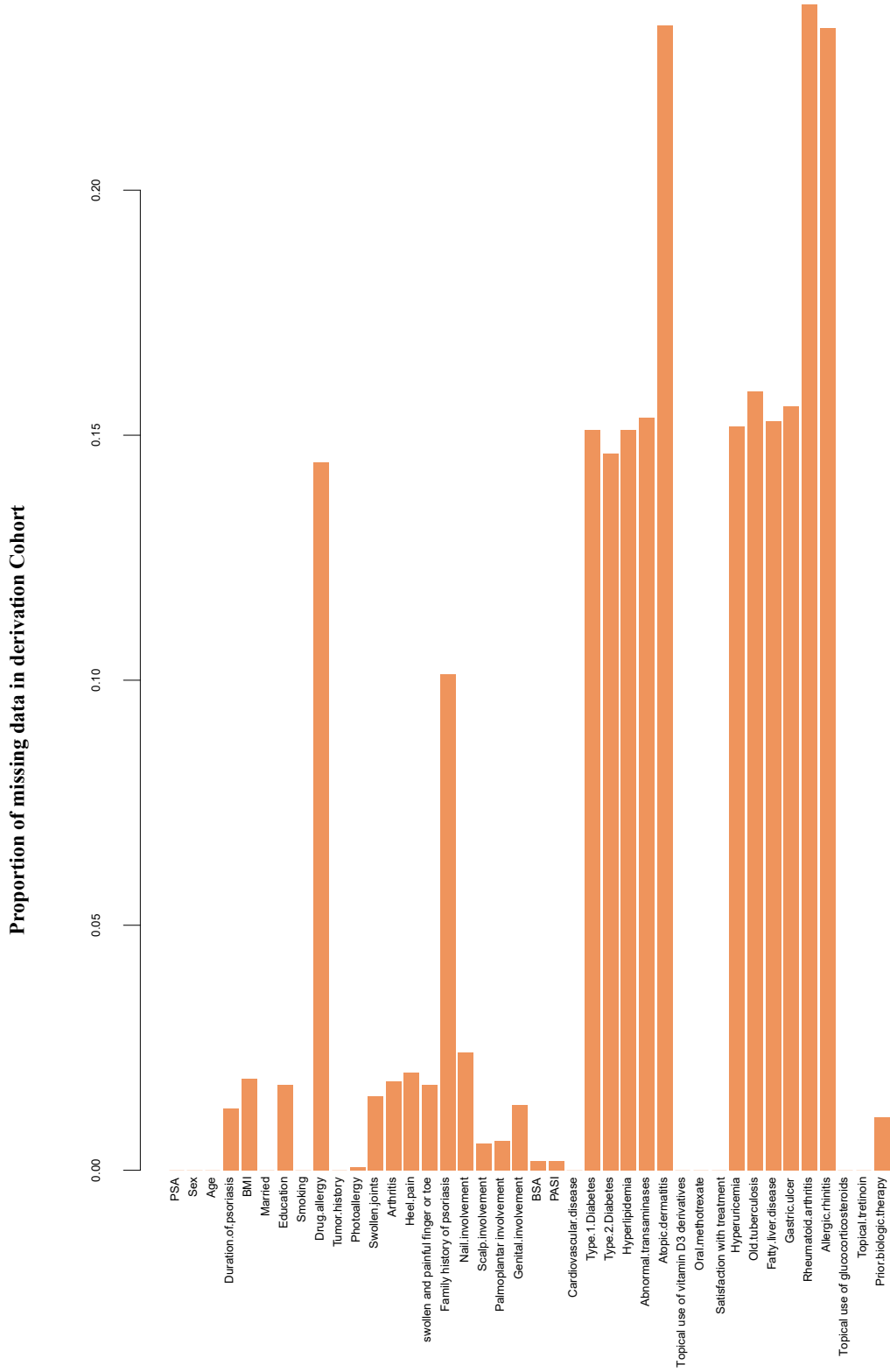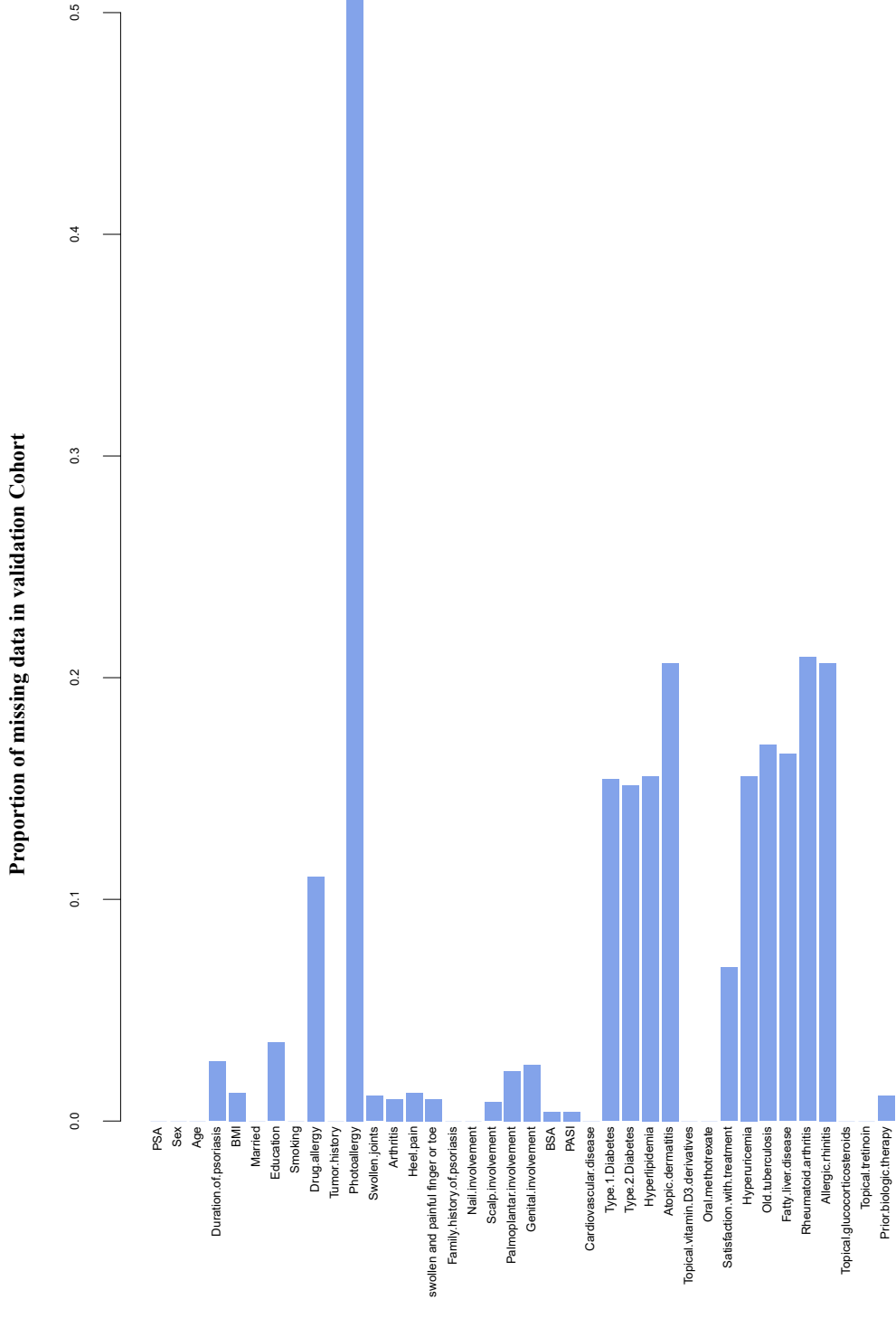

Supplement: Supplementary Figure 1 — Proportion of Missing Data in Derivation Cohort and Validation Cohort. [file Image1.pdf]

Fig. S2. Receiver Operator Characteristic (ROC) curve and Calibration Curve in Validation

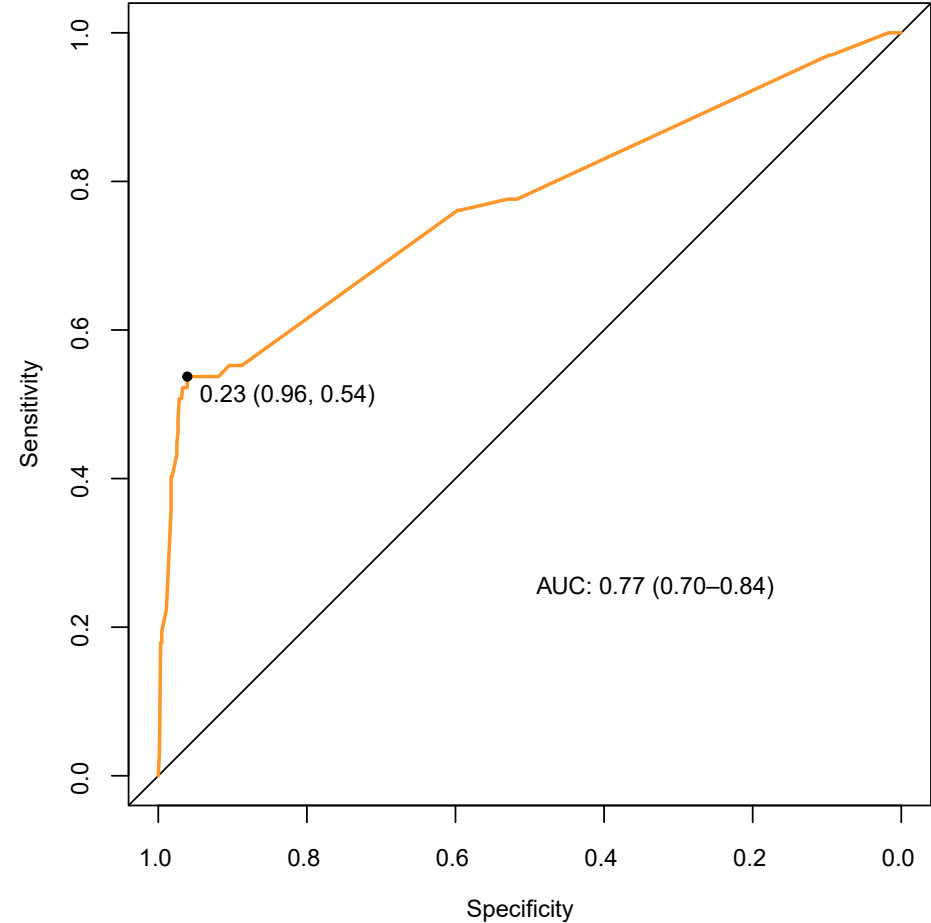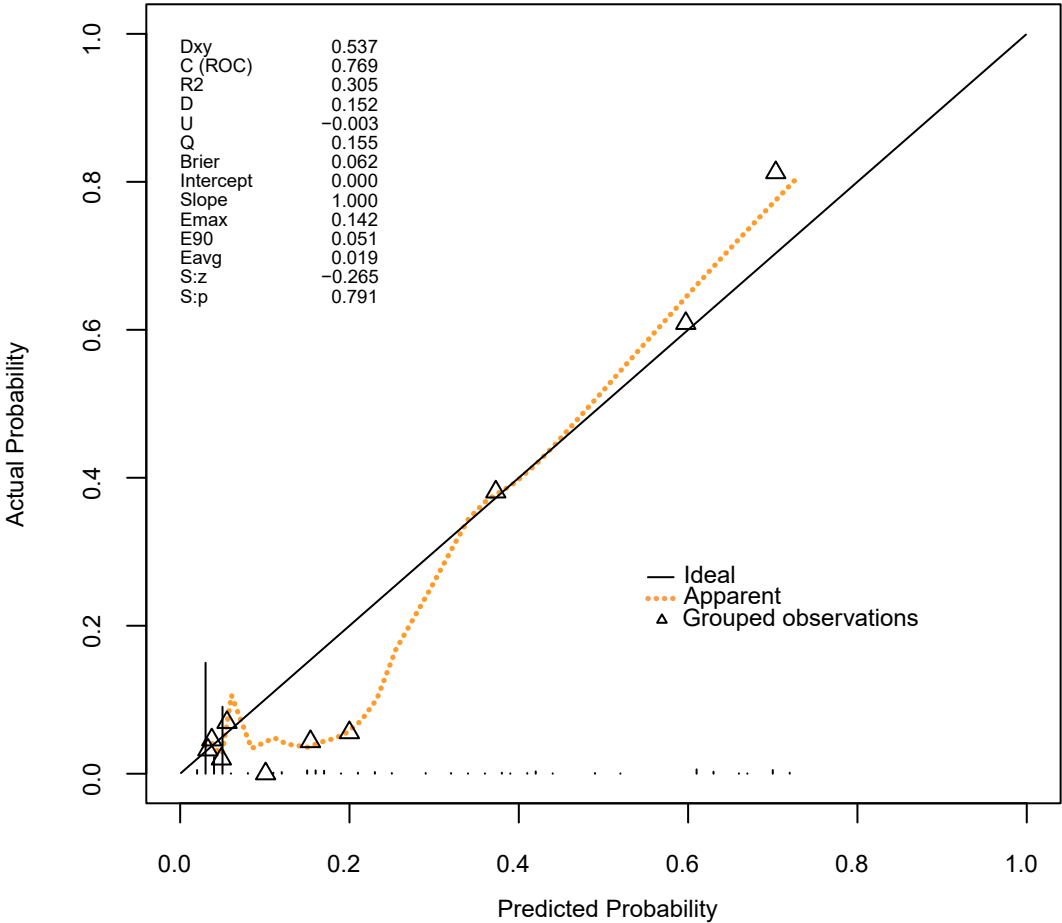

Supplement: Supplementary Figure 2 — Receiver Operator Characteristic (ROC) curve and Calibration Curve in Validation Cohort. [file Image2.pdf]
