# Supplementary material for: Early detection of psoriatic arthritis in patients with psoriasis: construction of a multifactorial prediction model
Source: Front Immunol. 2024 Dec 11;15:1426127. doi: 10.3389/fimmu.2024.1426127 (PMC11668630; doi:10.3389/fimmu.2024.1426127)
Supplement: Supplementary file 4 [file Table2.docx]

**Table S2. Demographics and Clinical Characteristics of Patients in Validation Cohort**

| **Variables** | **Total (n = 707)** | **PsA-yes (n = 67)** |
| --- | --- | --- |
| **Sex, n (%)** |  |  |
| Female | 252 (36) | 32 (48) |
| Male | 455 (64) | 35 (52) |
| **Age, Median (Q1, Q3)** | 38 (31, 51) | 42 (34.5, 54) |
| **Duration of psoriasis, Median (Q1, Q3)** | 7 (2, 17) | 10 (3, 20) |
| **BMI, Median (Q1, Q3)** | 24.11 (21.46, 26.72) | 23.71 (21.61, 26.01) |
| **Married, n (%)** |  |  |
| Yes | 513 (73) | 56 (84) |
| No | 194 (27) | 11 (16) |
| **Education, n (%)** |  |  |
| Junior high | 199 (29) | 16 (24) |
| Senior high | 282 (41) | 32 (48) |
| Undergraduate | 201 (29) | 18 (27) |
| **Smoking, n (%)** |  |  |
| No | 489 (69) | 49 (73) |
| Yes | 218 (31) | 18 (27) |
| **Drug allergy, n (%)** |  |  |
| No | 591 (94) | 53 (87) |
| Yes | 38 (6) | 8 (13) |
| **Tumor history, n (%)** |  |  |
| No | 703 (99) | 66 (99) |
| Yes | 4 (1) | 1 (1) |
| **Photoallergy, n (%)** |  |  |
| Yes | 75 (23) | 7 (17) |
| No | 248 (77) | 34 (83) |
| **History of unexplained swollen joints, n (%)** |  |  |
| No | 620 (89) | 29 (45) |
| Yes | 79 (11) | 36 (55) |
| **History of arthritis, n (%)** |  |  |
| No | 633 (90) | 33 (51) |
| Yes | 67 (10) | 32 (49) |
| **History of unexplained heel pain, n (%)** |  |  |
| No | 646 (93) | 47 (73) |
| Yes | 52 (7) | 17 (27) |
| **History of unexplained swollen and painful finger or toe, n (%)** | |  |
| No | 644 (92) | 36 (55) |
| Yes | 56 (8) | 29 (45) |
| **Family history of psoriasis, n (%)** |  |  |
| No | 102 (16) | 7 (11) |
| Yes | 545 (84) | 54 (89) |
| **Nail involvement, n (%)** |  |  |
| No | 537 (78) | 37 (55) |
| Yes | 149 (22) | 30 (45) |
| **Scalp involvement, n (%)** |  |  |
| No | 259 (37) | 22 (33) |
| Yes | 442 (63) | 45 (67) |
| **Palmoplantar involvement, n (%)** |  |  |
| No | 559 (81) | 44 (66) |
| Yes | 132 (19) | 23 (34) |
| **Genital involvement, n (%)** |  |  |
| No | 620 (90) | 56 (84) |
| Yes | 69 (10) | 11 (16) |
| **BSA, Median (Q1, Q3)** | 15 (5, 34) | 11.35 (3.25, 30) |
| **PASI, Median (Q1, Q3)** | 7.85 (3, 16.65) | 7.6 (2.73, 13.65) |
| **Cardiovascular disease, n (%)** |  |  |
| No | 674 (95) | 62 (93) |
| Yes | 33 (5) | 5 (7) |
| **Type-1 Diabetes, n (%)** |  |  |
| No | 596 (>99) | 53 (96) |
| Yes | 2 (<1) | 2 (4) |
| **Type-2 Diabetes, n (%)** |  |  |
| No | 585 (98) | 52 (98) |
| Yes | 15 (2) | 1 (2) |
| **Hyperlipidemia, n (%)** |  |  |
| No | 595 (>99) | 52 (98) |
| Yes | 2 (<1) | 1 (2) |
| **Atopic dermatitis, n (%)** |  |  |
| No | 560 (>99) | 53 (>99) |
| Yes | 1 (<1) | 0 (<1) |
| **Topical use of vitamin D3 derivatives, n (%)** | |  |
| No | 532 (75) | 55 (82) |
| Yes | 175 (25) | 12 (18) |
| **Oral methotrexate, n (%)** |  |  |
| No | 659 (93) | 59 (88) |
| Yes | 48 (7) | 8 (12) |
| **Satisfaction with treatment, n (%)** |  |  |
| Highly satisfied | 58 (9) | 7 (11) |
| Satisfied | 189 (29) | 16 (24) |
| Ordinary | 297 (45) | 28 (42) |
| Dissatisfied | 97 (15) | 14 (21) |
| Extremely dissatisfied | 17 (3) | 1 (2) |
| **Hyperuricemia, n (%)** |  |  |
| No | 595 (>99) | 53 (>99) |
| Yes | 2 (<1) | 0 (<1) |
| **Tuberculosis, n (%)** |  |  |
| No | 586 (>99) | 52 (>99) |
| Yes | 1 (<1) | 0 (<1) |
| **Fatty liver disease, n (%)** |  |  |
| No | 589 (>99) | 52 (>99) |
| Yes | 1 (<1) | 0 (<1) |
| **Rheumatoid arthritis, n (%)** |  |  |
| No | 557 (>99) | 53 (>99) |
| Yes | 2 (<1) | 0 (<1) |
| **Allergic rhinitis, n (%)** |  |  |
| No | 559 (>99) | 53 (>99) |
| Yes | 2 (<1) | 0 (<1) |
| **Topical use of glucocorticosteroids, n (%)** | |  |
| No | 406 (57) | 34 (51) |
| Yes | 301 (43) | 33 (49) |
| **Topical use of tretinoin, n (%)** |  |  |
| No | 635 (90) | 62 (93) |
| Yes | 72 (10) | 5 (7) |
| **Prior biologic therapy, n (%)** |  |  |
| No | 624 (89) | 59 (88) |
| Yes | 75 (11) | 8 (12) |

**Abbreviations:** BMI: body mass index; BSA: body surface area; PASI: psoriasis area and severity index; PsA: psoriatic arthritis; PsA-no: Psoriatic patients who were not diagnosed with psoriatic arthritis; PsA-yes: Psoriatic patients who were diagnosed with psoriatic arthritis
